# NSAID intolerance: it is not always what it seems

**DOI:** 10.1186/2045-7022-4-S3-P7

**Published:** 2014-07-18

**Authors:** Tatjana Pecaric Petkovic, Werner J Pichler, Oliver Hausmann

**Affiliations:** 1 Department of Rheumathology, Allergology and Clinical Immunology, Switzerland; 2 Inselspital, University Hospital of Bern, Department of Rheumatology, Allergology and Immunology, Berne, Switzerland; 3 Löwenpraxis, Zurich, Switzerland

## 

Case A: 42 year old female patient without asthma or polyposis nasi presented with palmar and plantar pruritus, urticaria, facial angioedema and dyspnea on two occasions: 2 and 4 hours after the intake of two structurally independent NSAIDs (diclofenac 75 mg, mefenamic acid 500 mg). In both instances, pantoprazole, a proton pump inhibitor (PPI), was prescribed for gastric protection. A non-allergic NSAID intolerance was suspected and avoidance of all classical cyclooxygenase (COX)-1 inhibitors recommended. Regardless, the patient's husband insisted on further allergological work-up remembering one unproblematic intake of mefenamic acid (500 mg) between the two events. Surprisingly, scratch testing was positive for pantoprazole only. This rare sensitization could be confirmed in basophil activation test (BAT). On further questioning the patient remembered that she forgot to take her pantoprazole co-medication when she transiently tolerated the mefenamic acid. The patient refused a provocation test with an alternative PPI. Due to their close structural relationship avoidance of all PPI was recommended. No general restriction for NSAIDs was necessary, and mefenamic acid was tolerated again. For long-term non-irritating anti-inflammatory pain treatment a COX-2 inhibitor (etoricoxib 30 mg) was advocated and well-tolerated.

## Conclusion

Intolerance to NSAIDs is frequent, but not all presumably "clear" cases are a classical NSAID intolerance, especially if the co-medication is known to cause anaphylaxis as well. This case supports the increasing numbers of reports showing PPI intake intolerance. A typical "pseudoallergic" clinical presentation alone does not exclude a true IgE-mediated allergy to a seemingly harmless co-medication. Thus, in patients with the frequent combination of NSAID and PPI a scratch test with the incriminated PPI is advisable. BAT can help to substantiate less reliable and reproducible skin test methods, like scratch test and is also useful to identify an alternative PPI.

